# Sharing lessons learned from COVID-19 vaccine introductions: a global community forum for countries

**DOI:** 10.3389/fpubh.2024.1376113

**Published:** 2024-05-14

**Authors:** Jenny Anne Walldorf, Cindy Chiu De Vazquez, Ana Carolina Barbosa De Lima, Bruce Struminger, Amy Groom, Lauren Burke, Landry Ndriko Mayigane, Diana Chang Blanc, Liviu Vedrasco

**Affiliations:** ^1^Department of Immunization, Vaccines, and Biologicals, World Health Organization, Geneva, Switzerland; ^2^Department of Health Security Preparedness, World Health Organization, Geneva, Switzerland; ^3^ECHO Institute, University of New Mexico, Albuquerque, NM, United States

**Keywords:** immunization, COVID-19, emergencies, program evaluation, community of practice

## Abstract

To optimize the efficient introduction and deployment of COVID-19 vaccines across the globe during the COVID-19 pandemic, in April 2021 WHO launched a new process and tools for countries to rapidly review the early phase of countries’ COVID-19 vaccine introduction. This methodology is called the COVID-19 vaccination intra-action review, also known as mini COVID-19 vaccine post-introduction evaluation (mini-cPIE). As of November 2022, 46 mini-cPIEs had been conducted. In collaboration with Project ECHO, WHO convened and facilitated real-time experience sharing and peer-learning among countries following their mini-cPIEs through a virtual global real-time learning forum. This five-session clinic series was attended by 736 participants from 129 countries. Based on post-session feedback surveys, when asked about the utility of the sessions, half of the participants said that sessions led them to review national guidelines and protocols or make other changes to their health systems. The post-series survey sent following the end of the clinic series showed that at least eight countries subsequently conducted a mini-cPIE after participating in the clinics, and participants from at least nine countries indicated the experience shared by peer countries on the clinic largely benefited their COVID-19 vaccine introduction and deployment. In this article, we highlight the benefits and importance of creating a global experience-sharing forum for countries to connect and share pertinent learnings in real-time during an international public health emergency. Moving forward, it is critical to foster a culture of individual and collective learning within and between countries during public health emergencies, with WHO playing an important convening role.

## Introduction

The World Health Organization (WHO) recommends that countries conduct a post-introduction evaluation (PIE) 6–12 months following the introduction of a new vaccine (1) to help countries evaluate implementation and take corrective actions. A PIE aims to fully assess the progress of vaccine introduction and can be time and resource-intensive requiring in-person field visits at all levels of the immunization system (2, 3). The COVID-19 pandemic, however, necessitated the rapid vaccination of the entire global population (4). Countries had little time to conduct a full programme evaluation such as a PIE, especially during the early phases of COVID-19 vaccine introduction and deployment (5).

To address this need, WHO developed a new evaluation method by adapting the WHO COVID-19 intra-action review (IAR) for countries to conduct a simpler, shorter and more flexible version of the PIE process, coined as mini-cPIE (6–8). The mini-cPIE was developed in line with the National Deployment and Vaccination Plan for COVID-19 vaccines (NDVP) (9), COVID-19 vaccine post-introduction evaluation (cPIE) and COVID-19 Strategic Preparedness and Response Plan (SPRP) (10). The WHO guidance for conducting a COVID-19 IAR (11) was developed soon after the pandemic onset for key stakeholders to collectively identify practices and lessons learned to course-correct and improve the ongoing response (12). During the IAR, a facilitator guides group discussions, in-person or virtual, over a period of 2–3 days using trigger questions pre-selected to address country needs. Based on the findings, the stakeholders then collectively recommend short-and mid-to long-term follow-up activities.

As countries and partners began using the mini-cPIE, they were eager to learn about ongoing vaccination implementation challenges and emerging best practices to improve their own processes and coverage. With this goal in mind, WHO collaborated with Project ECHO (Extension for Community Healthcare Outcomes) to create a global virtual community of practice (vCoP) called the mini-cPIE clinic, consisting of virtual learning sessions for countries to share and learn from experiences in real-time with immunization peers. These were referred to as “clinics” henceforth. During clinics, countries that had recently conducted a mini-cPIE were invited to present their learnings and best practices to peers that were planning or considering conducting a mini-cPIE. This was followed by Q&A/discussion that allowed for direct connection between countries.

Five clinic sessions were conducted from July to December 2021 (13). Clinic design was informed both by a past WHO-ECHO vCOP series on broader COVID-19 immunization, and by Project ECHO’s 20 years of experience implementing the ECHO collaborative-learning model. This model supports low-dose, high-frequency, virtual case-based learning to promote timely peer-to-peer information sharing and collaborative problem solving. This learning approach has proved to be instrumental in improving health outcomes during COVID-19, in other public health emergency responses and as related to general continuing health professional education (14–19).

The objectives of the clinics were threefold: (1) to create a forum for countries having conducted a mini-cPIE to share their experiences and learnings with stakeholders and peer countries; (2) to cultivate a community of learning and collaborative problem solving for COVID-19 vaccine introduction; and (3) to provide opportunities for countries interested in conducting a mini-cPIE to receive practical tips and technical guidance. The intended audience of the clinics were country immunization representatives interested in conducting, planning to conduct, or who had conducted a mini-cPIE, as well as partners technically or financially supporting these activities.

In this article, we describe the clinic design and participants’ characteristics, challenges, lessons, and best practices shared by presenting countries, and the perceived value of the clinics among participants.

## Methods

### Clinic design, content, and promotion

Between July and December 2021, WHO hosted a series of monthly, 90-min clinics on the Zoom videoconferencing platform. Clinics began with two to three country presentations followed by facilitated discussion, presentations from experts on immunization topics of interest, questions and answers, and interactive polls for real-time feedback from participants. The clinics were designed to be interactive, with participating countries able to directly engage with and ask questions to presenting countries. All questions that were not addressed during the clinic due to time constraints were compiled and shared with all participants and posted on the TechNet-21 immunization community collaboration platform (13). To maximize accessibility and promote multilingualism, simultaneous interpretation was provided to and from English, French, Spanish, Russian, Arabic and Portuguese. To further encourage participant engagement, a Telegram instant messaging group was established to offer participants an additional opportunity to continue discussions and experience sharing asynchronously outside the clinics.

Over the course of the six-month series, the Ministries of Health of 10 countries from four WHO regions, the African (AFR), American (AMR), Eastern Mediterranean (EMR) and South-East Asia (SEAR) regions, accepted the invitation to virtually present on their experiences with recently conducted mini-cPIEs (Table 1). The first two clinics addressed the conduct of and general learnings from mini-cPIEs -- including successes, challenges, and lessons learned -- and covered all immunization programme areas defined in the COVID-19 National Deployment Vaccination Plan (NDVP). The format of the clinics evolved through the series based on participant feedback and immunization topics that arose during that period of the pandemic. For the final three clinics, the presenting countries were asked to focus on specific immunization programme themes pertinent to challenges observed during the COVID-19 vaccine introduction and deployment (Table 1).

**Table 1:** Overview of the virtual mini-cPIE clinic series hosted by WHO and ECHO project, July–December 2021.

Session No.	Session date	Session theme
1	28 July 2021	Global Overview and Country Experience Sharing (Bhutan, Gambia, Senegal)
2	21 September, 2021	Mini-cPIE Experience Sharing and Lessons Learned (Ghana, Uganda)
3	12 October 2021	Experience Sharing and Lessons Learned in Fragile States/ Humanitarian Contexts (South Sudan, Somalia)
4	23 November 2021	Promoting Vaccine Uptake - Unique Risk Communication and Community Engagement Approaches (Democratic Republic of Congo, Mozambique, WHO Regional Office for Europe)
5	14 December 2021	Inequities in COVID-19 Vaccination Uptake (Bolivia), Gender and the COVID-19 vaccine roll-out

Advocacy and outreach efforts were made to ensure the intended target audiences were aware of scheduled clinic sessions. These included engaging with WHO regional and country focal points for both immunization and health emergencies so that they would share information about upcoming clinics with relevant country-level counterparts. Participants were also invited to join the sessions relevant to their interests through email announcements, social media, and Project ECHO and TechNet-21 platforms. Pre-registration was required for attendance and open to the public. After each session, Project ECHO distributed session materials (i.e., recordings, presentations, and Q&A responses) along with announcements for the subsequent session. Session materials were also posted on the TechNet-21 collaboration platform.

### Data collection and analysis

Participant demographic data were collected upon registration via a unique Zoom link, including name, email, gender, area(s) of expertise, professional affiliation, geographic level (e.g., multi-country, national, sub-national), and country. Registration and attendance data from Zoom reports were linked by email address. The first session registration data did not include gender, area(s) of expertise, and region of work. That and a few incomplete responses explain discrepancies in the result’s profile data totals.

A link to an anonymous online post-session survey (Supplementary Appendix 1) was shared with all participants for the third, fourth and fifth clinics, with digital certificates of participation provided to participants who completed the survey. The survey asked participants about their knowledge of the session’s topic before and after; relevance of the session to their current work; balance of lecture and interactivity; and intention to use what was learned in their work. In addition, Project ECHO distributed a post-series survey via email in November 2022 (eleven months after the last session) to all program participants to solicit feedback about the entire clinic series, including qualitative examples of barriers to conducting mini-cPIEs and improvements to vaccine roll-out linked to series learnings (Supplementary Appendix 2). The University of New Mexico Health Sciences Institutional Review Board approved the evaluation (ID 20–469).

Chi-square and Fisher’s exact tests were used to compare results across dichotomized geography (multi-country versus single-country) and country income levels. The Wilcoxon signed-rank test was used to compare knowledge before and after sessions. Quantitative analyses were conducted in R version 2022.07.1. Open-ended text responses were coded systematically using NVivo 1.4.1.

## Results

### Characteristics of participants

From 28 July 2021 until 14 December 2021, 996 attendances were recorded over five clinics. Among these, 736 unique individuals from 129 countries across all six WHO regions participated in at least one clinic session (Table 2 and Supplementary Figure S1). The number of participants in each session ranged widely from 88 to 241. Most (*n* = 563; 76.5%) participants attended only one session and 23.5% (*n* = 173) attended two or more. More participants (*n* = 436; 59.6%) described themselves as working in a single country versus multiple countries (*n* = 296; 40.4%) (i.e., working at the regional or global level). More than half (59.2%) of participants were in low or lower-middle income countries. The most common areas of expertise among attendees were immunization (*n* = 244; 37.7%) followed by emergency preparedness (*n* = 149; 23.0%) and program management (*n* = 146; 22.5%) (Table 2). Participants attended an average of 1.4 clinics each. Participants representing a single country were less likely to attend multiple sessions than those working at the regional or global level (Table 2).

**Table 2:** Demographics of participants who attended the virtual mini-cPIE clinic series hosted by WHO and ECHO project, July–December 2021.

Demographics	Frequencies		Attended 2+ sessions		
	*n*	%	*n*	%	*p*-value
**Geographic level (*n* = 732)**					
Multi-country	296	40.4%	86	29.1%	0.004
Single-country	436	59.6%	87	20.0%	
**Gender (*n* = 639)**					
Female	306	47.9%	76	24.8%	0.256
Male	333	52.1%	96	28.8%	
**Expertise (*n* = 648)**					
Immunization	244	37.7%	74	30.3%	0.296
Emergency preparedness and response	149	23.0%	40	26.8%	
Programme management	146	22.5%	36	24.7%	
Other	109	16.8%	23	21.1%	
**Country-level income (*n* = 736)**					
Low	104	14.1%	101	23.2%	0.793
Low-middle	332	45.1%			
Upper-middle	111	15.1%	72	24.0%	
High	189	25.7%			

### Lessons learned and best practices shared by countries

Country case studies were presented by either government representatives or WHO immunization experts from Bhutan, The Plurinational State of Bolivia, Democratic Republic of Congo (DRC), Gambia, Ghana, Mozambique, Senegal, South Sudan, Somalia, and Uganda. Speakers shared the importance of strong political commitment to mobilize national resources, bilateral negotiations for donations to increase access to vaccines, and multi-sector engagement to mobilize human resources. Strong inter-sectoral collaboration contributed to successes in every thematic area. Leading by example, political and community leaders were able to build trust and improve vaccine uptake.

Immunization experts from countries highlighted their successes with key innovations. Bhutan described how the Prime Minister boosted public confidence by receiving the first and second doses of a heterologous regimen. Bhutan achieved 95% vaccine coverage for the first dose and more than 90% vaccine coverage for the second dose following the first national vaccination campaigns. The Gambia described using a “vaccine caravan” to improve vaccine uptake and engage remote communities. Ghana used an innovative yet cost-effective approach for authentication of vaccination status with metallic holograms on vaccination cards, as well as utilizing drones to deliver vaccines to hard-to-reach areas.

Challenges and proposed solutions were shared together, although sometimes these solutions highlighted additional difficulties. For example, Senegal successfully established a COVID-19 Adverse Event Following Immunization (AEFI) committee and investigated all serious AEFIs; however, with this success came the challenge of how the country was addressing the lack of free medical care for those experiencing serious AEFIs. Uganda addressed delays in deployment of funds to the operational level by emphasizing early and transparent communication with health workers to instill confidence and allow continued vaccination services during resolution of administrative problems. Somalia described challenges in the availability and distribution of doses due to short expiry dates and described forecasting tools used to improve vaccine management and distribution. Bolivia worked to address coverage disparities through review of uptake data disaggregated by geographic area, target population, age, and gender. In low uptake populations, Bolivia prioritized single-dose regimens to minimize the risk of drop-out and reduce vaccine access inequity.

Strong risk communication and social listening activities at subnational and local levels were described by several Ministries of Health as essential to successful COVID-19 vaccine deployment. South Sudan dispelled rumors by acting on behavior survey findings through media engagement, high-level advocacy meetings, radio programmes and jingles, especially when new variants of concern emerged and created a loss of public confidence in COVID-19 vaccines. In the session focusing on unique risk communication and community engagement approaches, the DRC described strategies to build confidence among high-risk populations through strong interpersonal communication of health workers, pre-registration and monitoring of vaccination status to allow health worker follow-up and engagement. Mozambique used multiple communication channels to create public demand for vaccines and regularly monitored and managed rumors through a technical working group and digital platform.

### Poll responses

During the clinics, polls gathered real-time feedback from attendees. Examples of polling questions used were, “What topics/themes related to COVID-19 vaccine roll-out would you like to see discussed in future mini-cPIE clinics?” and “In a few words, what risk communication and community engagement strategies have you seen that worked well to increase COVID-19 vaccine uptake?.” The polls, whose answers were visible to all, were successful in enabling quick sharing of perspectives from multiple country or partner representatives from the audience and stimulated further discussion on new topics (Supplementary Figure S2).

### Post-clinic feedback surveys

The response rate for post-session surveys was 11.0% (*n* = 117). In post-session surveys, respondents rated their post-session knowledge significantly higher than before the session (Supplementary Figure S3) (*p* < 0.001). Almost all survey respondents (*n* = 110; 94.0%) reported the “right” balance of didactic and interactive learning. Additionally, most (*n* = 110; 94.0%) would definitely or probably recommend the session to a colleague and 80.2% reported the session was extremely or very relevant to their work. Most respondents would “definitely” (*n* = 94; 80.3%) or “probably” (*n* = 17; 14.5%) use information from the session in their respective work. The most common way participants planned to use the information was to share with colleagues (*n* = 81; 69.2%) followed by general use (*n* = 73; 62.4%), looking up additional information (*n* = 63; 53.8%), and making guidelines, protocols, or other changes to health systems (*n* = 60; 51.3%). Almost one-third (*n* = 37; 31.6%) planned to change how they worked with patients or community members.

Qualitative comments from post-session surveys reinforced the quantitative findings (Table 3). Respondents found the knowledge and information shared in each session useful and noted plans to share with colleagues or stakeholders as well as using them to develop communication strategies for advocacy or community outreach. One respondent reported, “*I liked the ideas of evidence-based communication, analysis of community feedback to craft communication products that respond to community inquiries*, etc.” while another said, “*The information shared in the session increased my understanding of challenges in other contexts which also apply to the African region*.” Answers from respondents helped inform priority COVID-19 topics for future sessions, including vaccines, booster dose policies, vaccine misinformation and hesitancy, financing, and metrics. Respondents also appreciated peer-sharing: “*It was a great cross learning opportunity. The challenges [encountered] in [fragile, conflict-affected, and vulnerable] settings are similar but the way these are addressed is the key learning*.”

**Table 3:** Feedback from participants to the open-ended questions of the post-session survey following the virtual mini-cPIE clinic sessions hosted by WHO and ECHO project, November 2022.

Theme	Description	Number of responses
**Usefulness**	**Responses to open-ended survey question “What part of this session was most helpful to your learning?”**	**154**
General	“All” or “everything”	54
Knowledge and information	Knowledge and information gained from sessions, including sharing with colleagues or using to develop communication strategies	45
Other countries’ experiences	Specific or general experiences shared from other countries	39
Other	Discussion, interaction, and question and answer components of session; gender and equality topics	16
**Recommended improvements**	**Responses to open-ended survey question “How could this session be improved to make it a more effective learning experience?”**	90
Topics	COVID-19 specifics such as vaccines, vaccine confidence, reinfection, adherence to preventive measures, specific country experiences, finances, and metrics	54
More time	Requests for longer or more sessions	12
More interaction	Requests for more ways to interact during sessions	11
Other	Clearer slides shared with participants, need for language interpretation	13

(*n* = 227).

### Post-series survey

The response rate for the post-series survey was 5.4% (*n* = 40) (Table 4). Most respondents primarily worked in a single country characterized as low or low-middle income (*n* = 19; 63.3%). Fifteen respondents (37.5%) from eight countries mentioned that their countries began planning or conducting a mini-cPIE due to their participation in the clinic series. For 66.7% of those respondents (*n* = 10), participation in the series significantly helped inform planning, implementation, and follow-up. Planning or implementation of a mini-cPIE was more likely to be reported by low income or lower-middle income country respondents compared to high income or upper-middle income countries (*p* < 0.01; Supplementary Figure S4).

**Table 4:** Characteristics of post-series survey respondents and reported impact of the virtual mini-cPIE clinic series hosted by WHO and ECHO project, November 2022.

	Frequencies	
	*n*	%
**Demographics**		
Geographic level (*n* = 40)		
Multi-country	9	22.5%
Single-country	31	77.5%
Country-level income (*n* = 30)		
Low	9	30.0%
Low-middle	10	33.3%
Upper-middle	7	23.3%
High	4	13.3%
**Impact of the clinic series**		
Country began planning or conducted mini-cPIE due to participation (*n* = 40)		
Yes	15	37.5%
No	10	25.0%
Do not know	15	37.5%
Extent participation improved COVID-19 vaccine rollout (*n* = 40)		
A lot	18	45.0%
Some	11	27.5%
Do not know	8	20.0%
Not at all	3	7.5%

Nearly three quarters of respondents (72.5%, *n* = 29) affirmed that their participation in the clinic series had improved their countries’ COVID-19 vaccine introduction and deployment a lot or some amount (Table 4). The improvements shared by the respondents varied but referred to specific strategies learned during the clinics. For example, “*More vaccination posts were created to support community access and decongest health facilities. In addition, vaccination campaigns to de-stigmatize vaccines were held across the country physically and through media*.” Additionally, respondents mentioned adjustments to their national vaccination plans, new strategies for prioritization of high-risk populations, strengthening vaccination data management, and long-term integration of COVID-19 vaccination efforts within the national immunization and health system. Seven respondents shared barriers to using learnings from the series including immunization decision-making being outside their scope of work or scheduling conflicts to participate in more sessions.

## Discussion

WHO, recognizing the importance and urgency of real-time peer-learning, leveraged its global coordinating role to rapidly convene countries and partners in the clinics described in this article. WHO, in collaboration with Project ECHO, created a global virtual learning forum for countries to highlight and share with peer countries their respective challenges, solutions, and innovations experienced during their COVID-19 vaccine introduction and deployment. While Project ECHO had prior experience establishing virtual communities of practice related to clinical medicine topics (16–18), this method for real-time peer-to-peer virtual learning was first used for the global immunization community during the pandemic. At the start of the COVID-19 pandemic prior to the mini-cPIE clinic series, WHO and Project ECHO collaborated on the ACT Accelerator COVID-19 initiatives (19), and a second series that convened partners and practitioners to build global capacity in preparation for COVID-19 vaccination (14). Several studies have shown that virtual training can help equip healthcare professionals with knowledge and skills for effective vaccination deployment during critical times of the COVID-19 pandemic (20, 21). The mini-cPIE clinic series was novel as it not only showcased the use of the intra-action review process recommended by the International Health Regulations Emergency Committee (22) early in the pandemic, but it also leveraged the virtual multi-country platform for case-based learning including country-level case presentations and participation from national public health practitioners.

Use of a virtual platform was essential given pandemic restrictions on in-person convening but also greatly reduced costs for convening large audiences. The establishment of the virtual platform also enabled WHO to continually advocate on behalf of Member States for additional technical and financial support from partners and donors. Participants who responded to post-session surveys found the clinics valuable. Survey responses show the value of clinics for participants in several ways, including a self-reported significant increase in knowledge, a high likelihood of endorsing the clinics, and plans to use the newly acquired knowledge. Evidence from the post-series survey reinforces this value, particularly for low and middle-income countries, by supporting the planning or conducting a mini-cPIE. Perceived value is also reflected in the suggestions for improvement, including requests for more sessions, more topics, and more time for discussion and interaction. The fact that most attendees attended only one and not multiple sessions suggests that either attendees were interested in a specific country presentation or a vaccination topic, or they were busy and could only select specific sessions to attend. Some participants may have taken advantage of the option to watch recordings of the sessions in addition to attending live.

Participant feedback was used to adapt clinic content to be most relevant and useful. For example, one of the clinics was dedicated to COVID-19 vaccine introduction and deployment in Fragile States/humanitarian contexts (13). The importance of strong risk communication strategies and community engagement was mentioned in multiple clinics, and given common challenges in this area, participants also expressed interest in having a focused session on the topic. This became the theme for session four (13), where presenters and participants shared regional and context-specific challenges and solutions. Global expert and audience contributions touched on key learnings that have since been documented in the literature (23, 24) including the importance of just-in-time risk-communication training and evidence-based community engagement approaches that rely on understanding the behavioral and social drivers to increase uptake and decrease hesitancy (25–28).

During the clinics, Q&A sessions generated considerable interest among participants, especially in the use of innovative approaches to increase vaccine coverage. Although it is encouraging to learn of the many emerging innovations borne of the COVID-19 crisis, best practice still requires that innovations be piloted and evaluated before scale-up, to assure that the innovative initiatives are beneficial, cost-effective, locally appropriate and sustainable. Equally, documentation of the impact of different approaches used by countries has been important to inform and optimize their use in the longer term for COVID-19 vaccine deployment, for other emergency vaccine responses or for routine vaccination programs (29).

In addition to innovation, countries described and discussed how best practice vaccination strategies from established childhood (e.g., polio, measles) and adult (e.g., influenza) immunization programmes were used to improve COVID-19 vaccination uptake. For example, strong outreach or mobile immunization sessions were described as crucial in many settings to reach priority populations. This finding aligns with literature documenting the use of outreach and mobile vaccination units to promote equity and reach underserved populations both prior to (30, 31) and during the pandemic (32–34). Moreover, enhanced engagement with private sector health providers expanded access to non-traditional or new vaccination sites. Strategic private sector engagement has been described elsewhere as an effective strategy for mass health emergency vaccination (35). During the pandemic, working collaboratively with different sectors in a whole-of-society approach was also highlighted in the global analysis of IARs conducted by WHO (36).

Established practice for one country might be innovation for another. Countries shared the mosaic of digital strategies reported subjectively to improve aspects of their vaccination programs: virtual training platforms; digital data management tools for tracking vaccine supply and distribution; electronic vaccination registries; electronic vaccination cards with sophisticated methods for authentication; digital platforms and social media for social mobilization, monitoring rumors, and infodemic management. Peer-to-peer learning on these practices allowed clinic participants the opportunity to seek support from country representatives or global partners to develop in-country capacity for the real-world implementation of evidenced-based strategies for digital applications across the immunization system from vaccine supply to delivery to monitoring to communications and community engagement. Evidence was beginning to accrue on use of these strategies prior to the pandemic (37, 38) but has expanded greatly with the pressures of the COVID-19 vaccine rollout (39, 40).

The development of this COVID-19 vaccination IAR (mini-cPIE) toolkit and the clinics initiated a new and collaborative process between the Health Security Preparedness Department and the Department of Immunization, Vaccines and Biologicals at WHO. The development process provided opportunities for relevant stakeholders and end-users at all organizational levels (global, regional, country) to provide feedback. WHO’s collaboration with Project ECHO’s academic team leveraged their global vCOP and interactive webinar production experience and added a new perspective for achieving the right balance of information sharing and peer-to-peer learning on global COVID-19 immunization. WHO often encourages countries to adopt a multi-sectoral, whole-of-government and whole-of-society approach to emergency preparedness and response. In this clinic series, WHO also followed this model from the design to the implementation.

Our analysis is limited by the fact that the response rates to the post-session and the post-series surveys were relatively low and cannot be generalized to all participants. For the post-series survey, this is likely related to the delay of almost a year after the completion of the clinics before the survey was conducted. It is possible that participants most likely to respond were those who experienced the greatest benefit. Regardless, the feedback provided by respondents indicates that the series may have prompted at least eight countries to conduct mini-cPIEs and many more felt the material shared led to positive change in practice.

## Conclusion and future directions

The creation of this virtual global real-time learning forum allowed countries to connect and share their COVID-19 vaccine introduction and deployment challenges and successes during an acute global emergency. A similar approach can be used to share pertinent topics beyond vaccination in real-time in future public health emergencies. The mini-cPIE clinics have also served as a springboard for a subsequent community of learning addressing lessons learned on COVID-19 immunization practices beyond the mini-cPIE (41). Moving forward, it is critical to foster a culture of individual and collective learning within and between countries during a public health emergency to help accelerate the sharing of new knowledge, promising practices, and real-world implementation of evidenced based practices, with global health agencies such as WHO playing an important facilitating role.

## Data availability statement

The raw data supporting the conclusions of this article will be made available by the authors, without undue reservation.

## Author contributions

JW: Conceptualization, Data curation, Investigation, Methodology, Supervision, Writing – original draft, Writing – review & editing, Resources. CC: Conceptualization, Data curation, Investigation, Methodology, Writing – original draft, Writing – review & editing. AB: Data curation, Formal analysis, Methodology, Visualization, Writing – original draft, Writing – review & editing. BS: Conceptualization, Methodology, Project administration, Resources, Supervision, Writing – review & editing. AG: Data curation, Writing – original draft, Writing – review & editing. LB: Methodology, Project administration, Writing – review & editing. LM: Conceptualization, Funding acquisition, Project administration, Resources, Writing – review & editing. DC: Conceptualization, Resources, Supervision, Writing – review & editing. LV: Conceptualization, Funding acquisition, Project administration, Resources, Supervision, Writing – review & editing.
