# Supplementary material for: Sharing lessons learned from COVID-19 vaccine introductions: a global community forum for countries
Source: Front Public Health. 2024 May 14;12:1376113. doi: 10.3389/fpubh.2024.1376113 (PMC11130350; doi:10.3389/fpubh.2024.1376113)
Supplement: Supplementary file 1 [file Image_1.pdf]

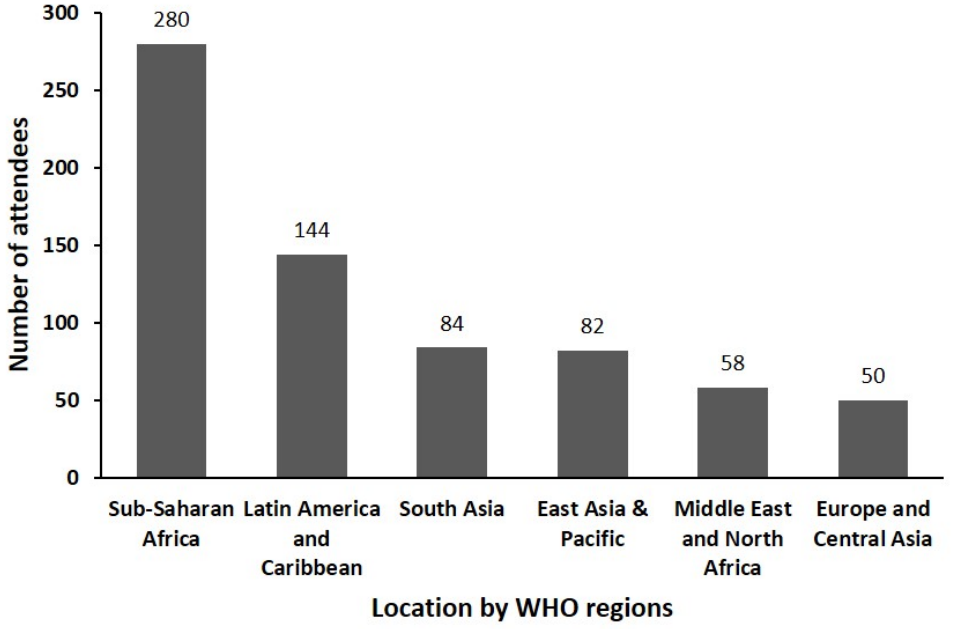

Supplemental Figure 1. Locations of attendees by WHO regions during the virtual mini-cPIE clinic series hosted by WHO and ECHO project, July-December 2021.

A. Map of where attendees were joining from for one of the clinic sessions.

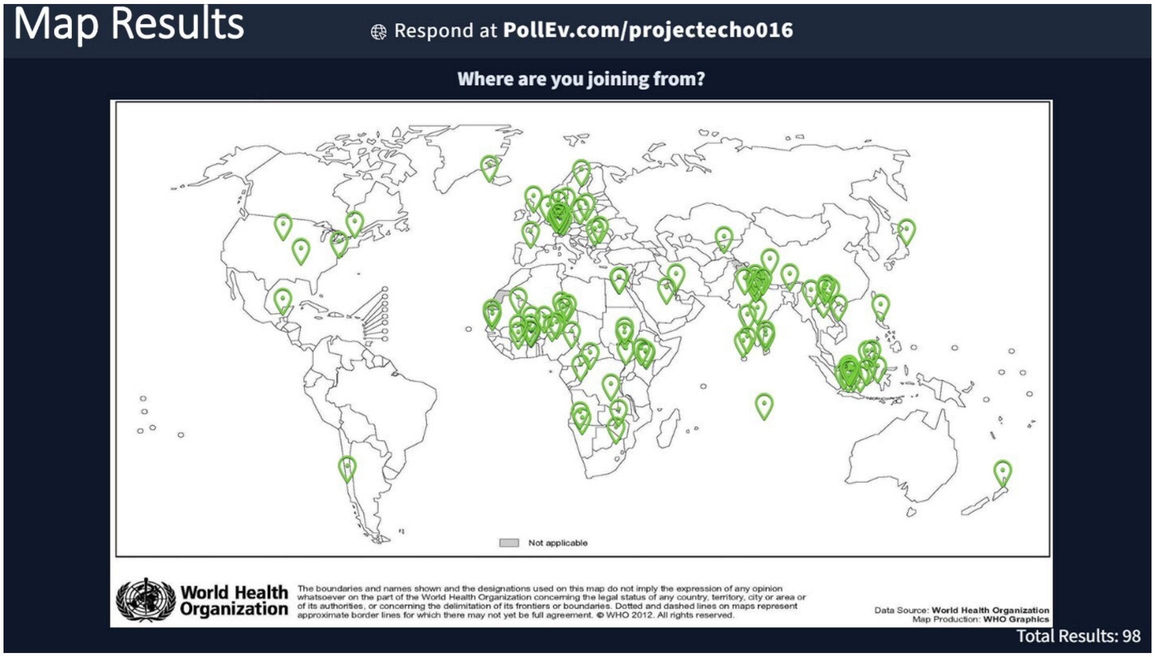

B. Real-time responses from participants to one of the interactive questions.

In humanitarian settings, what strategies have you found to be the most successful for reaching "populations of concerns" (e.g. IDP's, non-government controlled insecure areas, etc.)?

- Working with partners e.g UNHCR
- Working with NGOs
- For conflict areas, the hit and run approach for the safety of the teams
- Working with humanitarian NGOs and negotiating with groups in control of those areas for access
- For Cambodia, one dose strategy for the bordering provinces at least districts with 3 countries due to high population movement
- Integrated Mobile Medical Camps with COVID-19 vaccination in marginalized population
- Consider the target in the NDVP with microplaning at subnational area
- Mobile and outreach
- Training individuals from among these population of concern for enhancing advocacy works well
- Developed microplan and estimated TPs. J & J prioritize in HTR, IDPs etc
- Pop up vaccine site appear in the various districts
- Use of community volunteers in interpersonal communications
- Local vaccine distribution plans
- national policy and action plan
- Media; information session
- Mobile team
- Mobile Out reaches
- Outreach vaccination through mobile teams

Supplemental Figure 2. Examples of interactive activities during the virtual mini-cPIE clinic series hosted by WHO and the ECHO project, July-December 2021.

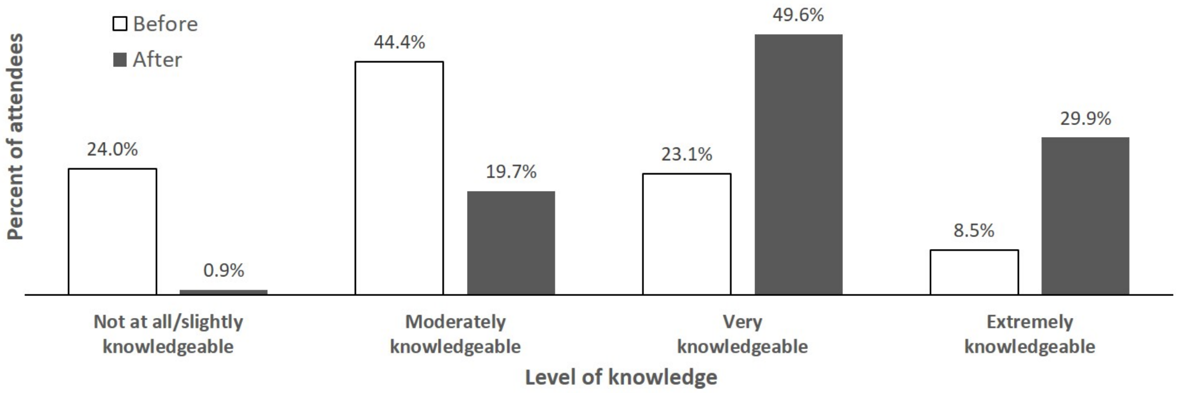

Supplemental Figure 3. Self-reported knowledge of session topic before and after the virtual mini-cPIE clinic series hosted by WHO and the ECHO project, July-December 2021 (post-session survey respondents, n=117).

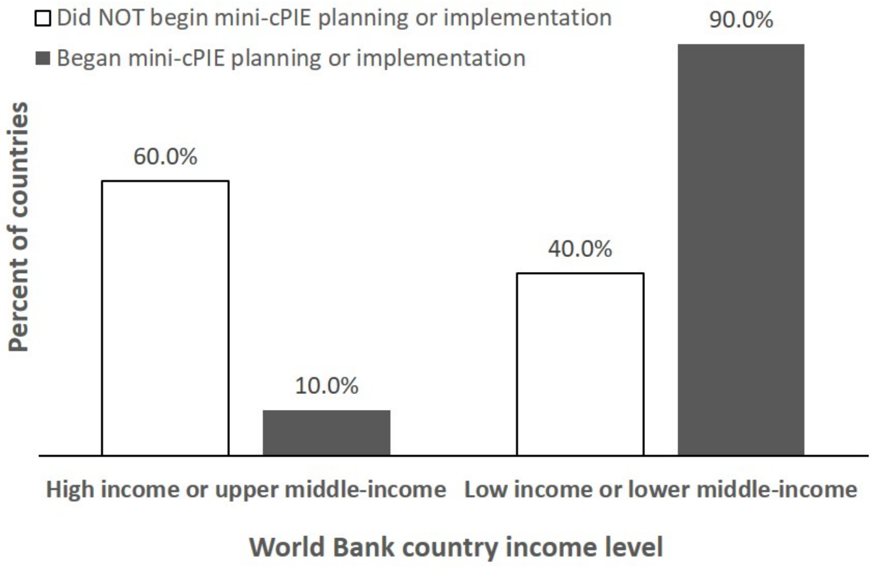

Supplemental Figure 4. Participants who indicated their countries began planning or conducting a mini-cPIE as a result of participating in the virtual mini-cPIE clinic series hosted by WHO and the ECHO project, July-December 2021 (post-series survey respondents, n=20).
